# A 5000-year overview of the history of pain through ancient civilizations to modern pain theories

**DOI:** 10.1097/PR9.0000000000001241

**Published:** 2025-04-03

**Authors:** Matthieu Vincenot, Pierrick Poisbeau, Nikolas Morel-Ferland, Geneviève Dumas, Guillaume Léonard

**Affiliations:** a CIUSSS de l'Estrie-CHUS, Research Center on Aging, Sherbrooke, Quebec, Canada; b Faculty of Medicine and Health Sciences, Université de Sherbrooke, Sherbrooke, Quebec, Canada; c Cognitive and Adaptive Neuroscience Laboratory, Centre National de la Recherche Scientifique, Université de Strasbourg, Strasbourg, France; d Department of History, Faculty of Letters and Social Sciences, Université de Sherbrooke, Sherbrooke, Quebec, Canada; e Université de Sherbrooke, Faculty of Medicine and Health Sciences, School of Rehabilitation, Sherbrooke, Quebec, Canada

**Keywords:** History, Humoral theory, East middle ages, Antiquity, Modern theories of pain

## Abstract

Pain and its management have been a predominant issue since the dawn of humanity. Pain has been the subject of much controversy and has constantly evolved across societies. The objective of this review is to trace the historical evolution of the concept of pain through the ages and to attempt to understand how modern theories of pain represent a legacy of ancestral knowledge passed down from culture to culture. We conducted a comprehensive review of primary and secondary sources across 6 major historical periods, including Pre-history, Antiquity, the Middle Ages, the Renaissance, the Modern, and Contemporary eras, using academic databases, specialized libraries, and historical archives. Results shows that during ancient civilizations, the understanding of pain oscillated between religious beliefs and medical advances. Antiquity societies made significant contributions to the understanding of pain mechanisms and management. Contrary to popular belief, significant advances were made during the Middle Ages despite the important impact of religion on the era's conceptualization of pain. During the Renaissance, the influence of religion waned, and secular medicine made significant progress. The development of pain theories peaked in the 19th century with technological advances. Long considered an expression of internal suffering, pain has fascinated humanity throughout history. The way pain has been perceived, understood, and treated has changed greatly over the centuries. This historical scientific review allows us to keep in mind that the vision we have of pain in Western society is predominantly based on the concrete heritage of ancient civilizations.

## Abstract 2

The way pain has been perceived, understood, and treated has changed greatly over the centuries from a divine punishment to a fully-fledged entity.

## 1. Introduction

In 2020, the International Association for the Study of Pain revised its definition of pain for the first time since its publication 30 years ago. Originally defined as an unpleasant sensory and emotional experience associated with actual or potential tissue damage, or described in terms of such damage,^72^ the definition of pain has been updated in 2021. It is now described as an unpleasant sensory and emotional experience associated with or resembling actual or potential tissue damage.^73^ This recent modification is an example of how the concept of pain can evolve to take into consideration new physiological knowledge and the evolution of societies. Although pain is a negative and unpleasant experience, it is an essential aspect of life. Its primary function can be compared with that of an alarm, enabling our organism to recognize threats, and adapt our behavior and learning accordingly. Above all, pain is an intimate experience, unique to each individual and evolves over the course of a lifetime.

Today, technology enables us to understand various aspects of pain, even the most personal ones. Brain imaging, for example, gives us a nearly direct view of the individual neural map of the pain response. This technology has not always been accessible; nevertheless, pain has been the subject of much philosophical, religious, and scientific questioning. Some theories, sometimes not so far away from what we have learned in the last few decades, have crossed eras and cultures. The concept of pain as arising from an imbalance of bodily fluids, as per Hippocrates' theory of the humors, has transcended numerous cultures and epochs, from Ancient Greece to the Renaissance. Examining the role of pain across diverse societies not only illuminates its historical development but also mirrors the functioning and values of those societies. How did the fall of the Western Roman Empire contribute to a return to the forgotten knowledge of ancient Greece in the late Middle Ages, some of which is still partly used today? The modern theories of pain, which developed in particular from the 19th century onwards, offer a glimpse into a distant cultural heritage dating back thousands of years.

As pain in inherently a cultural and societal construct, its role, perception, and understanding have naturally evolved alongside societal changes. The main purpose of this review, which we wanted to keep as narrative as possible, was to provide an overview of how pain has been understood and treated throughout history, from ancient civilizations to contemporary times. Such an endeavor can prove complex because no single characterization of pain can encompass the array of clinical, cultural, or theological definitions that have been in use at some point in written history. Historians focusing on the phenomenon of pain in the *longue durée* have, however, been able to circumvent such a semantic trap by broadening their understanding of the concept by focusing on the patient's private experience and its cultural significance for a given time and place.^2,9,63^ In doing so, this review also aims to highlight the important contribution of knowledge from ancient civilizations and the Muslim world to the development of the modern theories of pain.

## 2. Methods

**Figure 1.**
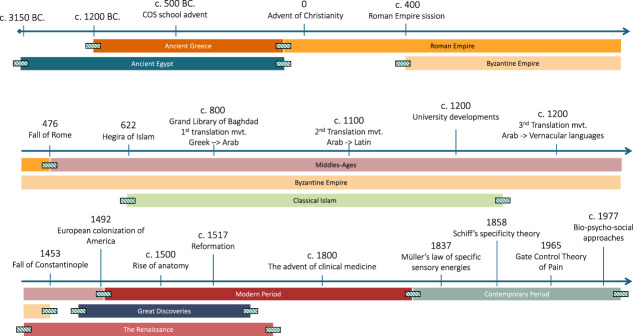
Timeline illustrating general historical landmarks. The passage from one historical period to another is a continuous process, devised a posteriori. The hatched rectangles on either side of the identified periods denote a margin of overlap.

We identified relevant primary sources (documents created during the period under study) and secondary sources (documents that analyze a primary source) relevant to 6 major historical periods: Pre-history, Antiquity, the Middle Ages, the Renaissance, the Modern, and Contemporary eras (Fig. 1). We relied on academic databases in the sciences and humanities, specialized libraries, and historical archives. We collected historical works, ancient medical treatises, manuscripts, and contemporary scientific publications that explore both the historical conception of pain and its evolution, encompassing perceptions, theories, and practices related to pain. The analysis of primary and secondary sources was limited to documents published in English or French. We performed a historiographical analysis of the documents collected, focusing on historical contextualization, comparison of medical discourses across different periods and cultures, and identification of sociocultural, religious, and philosophical factors influencing conceptions of pain. We also examined the methods of pain treatment and management used in different periods, considering medical advances, popular beliefs, and traditional practices.

## 3. Pain in early mankind: a matter of survival

### 3.1. A matter of survival

Alleviating pain has certainly been one of the main concerns of our species because its presence can lead to vulnerability and reduce chances of survival. Certain cave paintings depict gestures that can be interpreted as a form of pain management. Some are very primitive and show mammals licking wounds, whereas others are more elaborate and depict the use of tools to relieve suffering, up to and including the amputation of an injured and painful limb.^54^ The origin of the pain was probably considered esoteric for many civilizations, resulting from demons or malevolent spirits inside the body, until the spread of holistic medical theories in the Classical Antiquity. In most treatments, mystical powers were invoked with amulets or talismans, and occasionally involved tattooing or scarring of the skin.^80^ Recently, one of the world's earliest traces of cranial surgery was discovered in northern Spain in a person suffering from an infection of the mastoid.^23^ Archaeologists noted regeneration of the bones after surgery, indicating that the individual had survived the operation. This finding complements the numerous observations of rudimentary surgical procedures that were also performed.^42,50,95^ Early civilizations also used plants that had analgesic properties, as well as important nutritional functions. The earliest known evidence of this dates from around 3000 bc with the coca leaf, which was a potent painkiller and was used in various forms (ingested, dried, smoked, and as a poultice) by the peoples of pre-Columbian America.^77,80,88^ Recent data have also demonstrated that opium poppy has been present naturally since at least the middle of the sixth millennium in the Mediterranean region, where it was probably cultivated by pioneering Neolithic communities.^81^

### 3.2. Becoming a man

Pain, however, could also have different meanings. The numerous depictions of self-mutilation (tongue, ears, penis) in the material culture of precontact America suggest a different symbolism; pain could also represent a social rite of passage. By resisting pain, the individual proved their strength and worth. Such rituals still exist today; for instance, within Brazilian *Satere-Mawe* tribe, boys aspiring to manhood are tasked with placing their hands in a basket teeming with “bullet ants” (ie, *Paraponera clavata*), notorious for their excruciating stings.^8^

## 4. The place of pain in the ancient world

### 4.1. In the hands of the gods

The rise of urbanization in 3500 bc in Mesopotamia (formerly the actual Iraq and Syria for the most part) in the area known as the fertile crescent provides some of the earliest written records to shed light on the medical practices of the Ancient Near East. Archaeologists investigating the “Code of Hammurabi” have established that Old Babylonian society heavily regulated health care practices, going as far as to set up rates for specific surgeries.^86^ The most common categories of medical practitioners, the *āšipu* and the *asû*, played an important role in the civic and religious life of their society and contributed to the early development of diagnosis, prognosis, and therapy.^26^ Specifics symptoms, including pain, were associated with illnesses that were frequently perceived as omens sent by deities.^39^ The diagnostic handbook “When the āšipu goes to the house of the patient” (original title: “enūma ana bīt marşi āšipu il”) illustrates a high degree of awareness of common medical impairments that plagued patients, such as headaches, trauma, and infectious diseases.^29^

Near Mesopotamia, the Egyptian civilization viewed pain as a mystical and religious process. Demons, sent by the gods, entered the body through the orifices of the face, and physicians and priests were unsure as to how pain should be treated.^80^ Invocations to the god *Horus* and religious rituals were part of the essence of pain treatment, with the goal of driving away pain. Sneezing, vomiting, and urination were common and basic therapeutic measures.^80^ In parallel, innovative techniques were being developed. Electrotherapy, conducted with torpedo fish from the Nile river, was used to relieve headaches and joint pain.^80^ To limit the pain of circumcision, practitioners used a form of cryoanalgesia induced by a chemical reaction to numb the area before the procedure.^27^ In addition, numerous texts (Smith and Ebers papyrus from around 1500 bc) referenced the medicinal effects of hundreds of plants, including opium, in some surgical techniques.^59,70^

### 4.2. At the confluence of philosophy and medicine

Across the Mediterranean Sea, Classical Greece (around 510–323 bc) was one of the richest periods in physiological and philosophical advances in the field of pain. Indeed, some of the concepts that would later be presented as “innovative” in the 19th century were already addressed in Antiquity.

In the Iliad and the Odyssey, pain is presented as a divine punishment, with a wide range of pain semantics presented. These terms are not necessarily limited to bodily suffering and can convey emotional trauma, such as expressed by Ulysses' return to Ithaca in his Odyssey “I have suffered much, but at last, in the twentieth year, I am come back to my own country” (Odyssey, Book XXI, 203). The Greeks already distinguished between physical and moral pain (Table 1). Pain was personified through the Algos entity, representing the daughters of Eris, the goddess of discord. Treatments were de facto associated with divinatory cults.^3,31,82^

**Table 1:** Semantic of pain in ancient Greek language.

Words	Signification
Οδύνη (Odunê)	Specific and localized physical pain
άλγος (algos)	General pain
πονος (ponos)	Describes the notion of drudgery and suffering

However, the idea of a biological origin for pain was also gradually emerging. Alcméon of Crotone (c. 566–497 bc) was one of the first to see pain as directly related to the function of the brain, the center of the nervous system and intellect. Hippocrates of Kos (Table 2), whose medical legacy spans millennia, described pain as the result of an imbalance between the body fluids and considered the brain as the center of sensations. His ideas were carried out by his pupils, who refined and published them. The Hippocratic treaty entitled “On the nature of Man” (original title: “De Natura Hominis”), attributed by historians to his son-in-law *Polybu*s,^37^ brought forward the humoral theory. According to this paradigm, diseases were caused by a disturbance of 4 humors (blood, yellow and black bile, and phlegm); in such a system, pain relief strategies were primarily designed to return the body to a balanced internal state using various medical proceedings. This theory would shape the vision of pain for centuries to come.

In his last dialogue *Timaeus* (c. 358 bc), Plato also defined pain, conceptualizing it as an emotion that arises from intense and prolonged stimulus. This proposal gave rise to the theory of intensity. About 100 years later, *Herophilus* (c. 325–255 bc) and *Erasistratus* (c. 310–250 bc) provided anatomical evidence that the brain governs our thoughts and actions. They defined 2 neurological pathways: one pathway responsible for movement and another responsible for sensation. It be centuries before Charles Bell provided experimental evidence on this subject.^4,40^ Although these early scientists were partially correct, the role of the brain as the central organ of sensation was not the prevailing ideology: the vision of Aristotle (Table 2) still prevailed for many years and excluded the role of the brain in governing the passions of the soul (eg, pleasure and pain). According to Aristotelian thought, as proposed by Plato, the center of sensory perception—referred as the “common sensorium”—was believed to originate in the heart.^43^

**Table 2:** Some illustrious figures in the History of pain.

Hippocrates	Physician considered the “father of medicine.” His teachings emphasized clinical observation and the search for natural causes of disease, marking a crucial step in the evolution of medicine.	° −460 † −370
Aristotle	Aristotle was a Greek philosopher and student of Plato. His contributions covered a wide range of fields, from metaphysics to politics, biology, and physiology.	° −384 † −322
Galen	Physician, philosopher, and anatomist, he is considered one of the most influential medical practitioners of antiquity. His writings shaped Western medicine for over a 1000 years, emphasizing clinical practice and careful patient observation.	° 129 † 216
Avicenna	An eminent Persian scholar, he is considered one of the most influential figures in the history of Islamic medicine. His major work, “The Canon of Medicine,” has become a fundamental medical text.	° 980 † 1037
Ambroise Paré	A renowned surgeon (employed by monarchs). He introduced innovative methods for the treatment of war wounds. His contributions reduced patient suffering and paved the way for major advances in surgery.	° 1509 † 1590
René Descartes	Philosopher, mathematician, and scientist, he is considered one of the founders of modern philosophy. His work had a considerable influence, marking a break with medieval scholastic thought.	° 1596 † 1650
Charles Bell	Charles Bell was a Scottish surgeon, anatomist, and neurologist known for his discoveries in anatomy and physiology, particularly his elucidation of the distinction between sensory and motor nerves.	° 1774 † 1842
François Magendie	François Magendie was a French physician and physiologist. He held the first research chair in experimental physiology at the Collège de France, and initiated the first work in pharmacokinetics, with a particular interest in the nervous system.	° 1783 † 1855
Alfred Goldscheider	Trained as a neurologist, he is renowned for his important contributions to research into sensory pathways and cutaneous sensitivity.	° 1858 † 1935
Maximilian von Frey	An Austro-German professor of physiology, he is renowned for his work on mechanoreceptors, for which he developed a measuring instrument still in use today: Von Frey filaments.	° 1852 † 1932
John Bonica	An anesthesiologist, he is recognized as one of the pioneers of pain management and played a major role in establishing the specialty of pain medicine. Bonica founded the first pain clinic in the United States in 1947 and made major contributions to research into the mechanisms of pain.	° 1917 † 1994
Ronald Melzack	Trained as a psychologist, he is renowned for his groundbreaking work on pain, the “gate control theory” in 1965 (in collaboration with P. Wall). This theory has profoundly influenced modern understanding of pain and paved the way for new approaches to pain management.	° 1929 † 2019
Patrick D. Wall	A British physiologist and professor at the renowned Massachusetts Institute of Technology, he conducted in-depth research into pain mechanisms. With his colleague R. Melzack, he contributed to the famous gate control theory.	° 1925 † 2001

The Roman Empire's expansion became ever more imposing after Greece finally became a Roman province after its defeat at the Battle of Corinth (146 bc). Roman medicine inherited much of the knowledge acquired by the Greeks.^82^ For nearly 4 centuries, Aristotle's doctrine and the idea of religious origins of pain remained firmly embedded in the Roman medical world. A fruitful period of Roman medicine began in the first century ad with the work of Galen^3^ (Table 2). Galen insisted on the notion of a central and peripheral nervous system at the center of the sensory system.^64^ He recognized pain as an alarm signal that could be used to identify the origin of an injury.^64,80,96^ Galen presented pain as a disruption of continuity, or anything that was not in the natural course of things.^64,78^ Unfortunately, Galen's peers did not recognize his work, and Aristotle's philosophical conceptualization of pain would dominate Europe for many more centuries to come.

In the realm of treatment, botany was flourishing. *Pedanius Dioscorides* (c. ad 25–90), a pioneer in pharmacognosy, authored a work that remained influential until the Renaissance, and which comprised descriptions of nearly 1500 substances, including plants, minerals, and animal matter, lauded for their medicinal properties.^68^ Various preparations containing opium were commonly used to treat pain. Nonpharmacological treatments were also emerging, with recommendations advocating for the therapeutic benefits of electric currents. Scribonius Largus (c. 1–50 ad), a roman physician, recommended in his “Compounding of Drugs” (original title: “Compositiones medicamentorum”) placing torpedo fish on the foreheads of patients with headaches.^14,32^

As Egypt was under Roman domination (*Provincia Aegypti*) for several centuries, spanning from Octavian's conquest of the Ptolemaic kingdom in 30 bc to the Arab reconquest in 641, it is unsurprising that Egyptian pain management techniques were in use in Roman society during this period. It was not until the 19th century that the first theoretical model of pain explained the basis of this technique. Other ancient techniques, such as cold application and massage, which have been practiced since ancient Egypt,^3^ remain prevalent in contemporary therapeutic practices. Before their widespread social use by the Romans, hot baths and thermal baths were already used in Egypt and Greece and served a medicinal purpose.^36^

## 5. Middle ages and the renaissance

### 5.1. The advent of christianity

Contrary to popular belief, the Middle Ages, particularly in the Eastern world, were a fertile period in history marked by significant medical advancements, some of which were influenced by religion. It is important to acknowledge and appreciate that hygiene practices were more developed than we tend to believe, including for medical purposes.^90^ The Fall of the Western Roman Empire (476 ad) heralded the end of the ancient era; political fragmentation that ensued had a significant impact on the maintenance of previous infrastructures, including medical dispensaries managed by Romans. The conversion of Constantine the Great (312 ad) enabled the Christian religion to spread rapidly across Europe. As in other historical periods, the divine nature of pain persisted and evolved through this renewal of faith. Pain was believed to have both a punitive character, like Eve's punishment for the original sin, but also to be redemptive,^20^ because it was associated with martyrdom and the Christ's earthly journey in scripture. This attitude toward pain as a positive entity was termed *philopassianism*.^20^ Such a powerfully ingrained conception somewhat downplayed the utility of research investigating possible causes of illness as well as pain treatments.^90^

### 5.2. Eastern culture

Although scientific and medical progress was an incremental process in Europe, medicine made great strides in the Muslim world. After the fall of Rome, in an effort to appropriate knowledge, the works of the great authors of ancient Greece and Rome were translated into Arabic under the impetus of caliphs such as Harun Al-Rachid (765–809) and Al-Mamoun (830–894) around the ninth century. The House of Wisdom (*Bayt al-Ḥikmah*) in Bagdad, a scientific institution that welcomed scholars and physicians from all over the Eastern world played an important role in this translation movement.^1,52^ An important work of synthesis of ancient knowledge was done, resulting in original Arabic medicine. However, this translation movement has not been flawless and sometimes resulted in misunderstandings and mistranslations.^62,74^ Simultaneously, the health care system was undergoing significant change, with hospitals being reorganized and public funds allocated for their operation. An important culture of books and knowledge transmission was being established.^71^

*Abu Ali Al-Husayn Ibn Sina*, known in the West as Avicenna (Table 2), was an important figure in the Eastern world's medicine.^24^ He mainly investigated Galen's work, refining the studies in the “Canon of Medicine” (original title: “Kitab Al Qanûn fi Al-Tibb”), a Galen's book in which pain holds a significant place. Avicenna suggested that pain is not a disease in itself but rather the sign that something has occurred, whether on a physical level or in the balance of humors. He described the etiology of 15 different types of pain (of the 50 proposed by Galen), with specific words and adjectives. Avicenna's works were not introduced to the West until the 12th century, alongside other foundational medical texts from the Arab world, such as those of Al-Razi and Al-Kindi. This translation movement, which focused on scientific literature from Arabic and Greek into Latin, began in Southern Italy with Constantin the African's translations of Galenic texts. It later continued in Toledo, where a diverse group of international translators, including several Sephardi Jews, worked on translating not only Avicenna's *Canon* but also Galen's commentaries on Hippocrates. The influx of this new knowledge into the West sparked a thirst for learning, ultimately leading to the establishment of European universities, where medicine became one of the core faculties.

### 5.3. Medieval Europe

Avicenna had a lasting impact on medicine in Europe and inspired the pioneering work of physician Guy de Chauliac (1300–1368). De Chauliac's definition of pain in the “Great Surgery” (original title: “Chirurgia Magna”) in 1363 borrowed from Avicenna, describing it as “a sensation of contradictory qualities capable of causing pain, but sometimes associated with alterations that break or cut, stretch or abrade.”^93^ As for treatment, opium remained in vogue, but its sometimes-abusive use raised questions and concerns. It would not be until the end of the Middle Ages (15th century) that initial work on opium dosage would be done.^80^ Despite this, the theory of humors retained its presence and influence throughout the Middle Ages, resulting in limited anatomical studies. According to this theory, the circulation of bodily fluids is vital; upon death, fluid movement ceases, rendering human body dissection unnecessary.

The growing expansion of the Church brought along multiple medical advances through the necessity of translation. The Crusades—military expeditions initiated by the Pope to reconquer the holy lands taken by the Muslims—gave rise, around the 12th century, to a second translation movement around the 12th century^13,53^ that would give Europe renewed access to Antiquity and Arab medical knowledge. During the Middle Ages, Europe was battered by major epidemics, notably the plague. The exponential increase in the number of patients forced the health care system to adapt and evolve. The dispensaries previously located in monasteries were replaced by hospitals, urban structures under the responsibility of the State and no longer managed by the clergy.^19^ In parallel, the expansion of Islam into North Africa and Europe through the Iberian Peninsula and Southern Italy gave rise to major cultural and linguistic exchanges that enabled Western Europe to acquire various medical works. This effervescence paved the way for the creation of the world's first universities, where medicine was one of the main disciplines taught along with canon law and theology. The Salerno School of Medicine, one of the first in Europe, taught medicine based on ancient and Arab knowledge. Medical practice became regulated in several European countries, with requirements for training and authorization to practice.^91^ Gradually, the practice of medicine became increasingly restricted for ecclesiastics, favoring lay medical practice.

Medieval therapeutics can be divided into 3 levels of intervention on the body.^55^ The first level was diet. The second level was the use of pharmacology using plant, animal, and mineral substances. *Lodanum* and the *spongia somnifera* are 2 examples.^38^ The third level was surgical intervention, including the famous bloodletting. Bloodletting had been practiced since ancient times and remained a main method of treatment up until the late 19th century. The practice of bloodletting was tied to the Hippocratic humoral theory; its purpose was to cleanse the diseased blood that was the cause of various ailments so that it could be replaced naturally with new blood.^12^ In medieval times, most medical practitioners were men of faith from the clergy. However, blood was considered taboo, as Christians were prohibited from shedding the blood of their brethren. As a result, physicians limited the practice of bloodletting, which gradually found its way into the hands of barbers, who had not only the equipment but also the finesse and expertise (hence the term barber-surgeon).

### 5.4. Scientific renewal

The end of the Middle Ages was highlighted by 2 major forces: on one hand, competition rages to colonize the new world and its treasures; on the other hand, the population was being decimated by the plague epidemic ravaging the continent. Through this tumultuous period in European society, a new chapter was developing, with a newfound appreciation of classic culture that challenged conventional assumptions about arts and sciences, contributing to a renewed perception of the “individual.” Scholasticism (a medieval school of philosophy) lost some of its influence, but religion remained firmly entrenched in society (Galileo's condemnation by the Catholic Church for heresy in 1633 comes to mind). European physicians gradually incorporated new knowledge from Arabic medicine into their corpus. The idea of the dual somatic and psychological nature of pain was beginning to emerge, and many advances were made, especially regarding the nervous system. Leonardo da Vinci (1452–1519) defined nerves as tubular structures associated with the sensation of pain and touch. The evolution of anatomy culminated with Andreas Vesalius (1514–1564), anatomist and physician. Medical progress in the field of anatomy reached a milestone with his publication of the famous “On the Structure of the Human Body in 7 Books” (original title: “De Humani Corporis Fabrica Libri Septem”) published in 1543.

Great discoveries were made during the maritime explorations of Marco Polo, Vasco de Gama, and Christopher Columbus towards China, India and America which led to the development of a new pharmacopoeia (eg, *Cinchona, Cacao, Podophyllum, Ipecacuanha*, *Nicotiana tabacum*) and new analgesic practices.^68^

The last decades of the 16th century in Europe were perceived, even by contemporaries, as calamitous. Successive waves of the plague between 1563 and 1598 decimated the continent.^75^ At the same time, religious upheaval that stemmed from the Reformation led to a series of bloody conflicts. Centralization of political authority fueled wars of conquest, as enlisting men to fight was facilitated by a growing bureaucracy. The technical development of firearms created new injuries, and therefore, the need for new innovative treatments. Ambroise Paré (Table 2), one of the main figures of modern surgery along with Chauliac, played an important role during this period, first as a soldier, then as a master barber-surgeon in Paris. He became, in 1562, the official King's surgeon. Although perioperative pain was not considered at the time, Ambroise Paré crusaded for this cause. He was also one of the first to describe and take an interest in phantom pain.^79,79,87^

## 6. The revolution of modern times: between science and literature

### 6.1. The boy and the fire

It is difficult to review the 17th century without mentioning René Descartes (Table 2). Descartes, a philosopher and distinguished scientist in mathematics, physics, and physiology, embraced the idea that the brain occupies a central position in the sensory system, thus exerting significant influence over pain perception. In his work, he described nerves as a series of fine wires running through the skin and tissues connected to the brain. This idea is at the heart of the theory of specificity attributed to Descartes, which would later set the stage for the field of functional neuroanatomy in the 19th century. Descartes identified a structure in the diencephalon, the pineal gland, which he saw as the link between body and soul. Descartes also gained interest in phantom limb pain and theorized that some degree of nerve activity persisted in the missing limb, as if it were still present. Descartes will specify in his work “Meditations on First Philosophy” (original title: “Meditationes de Prima Philosophia”): “The pain of the (amputated) hand is not felt by the soul as being in the hand, but as being in the brain.”, introducing one of the first conceptions of central pain. Without direct reference to pain, Luigi Galvani (1737–1798) described in his “Commentary on the Effect of Electricity on Muscular Motion” (original title: “De Viribus Electricitatis in Motu Musculari Commentarius”) in 1791 the bioelectric properties of nerves and muscles in frogs. Electrophysiology will continue to develop over the centuries^92^ and will become one of the most widely used techniques for studying the nociceptive system.^33^

### 6.2. Rational thinking

The Age of Enlightenment (18th century) witnessed a profound change in European society, especially in France, where thinkers became preoccupied with the ideal of reason and universalism. The question of pain now arose for physicians and physiologists outside of the realm of sin, evil, or punishment. During the “medicine of the Enlightenment,” the inquiry into the utility of pain gained prominence as physicians scrutinized its origins and characteristics to facilitate diagnoses. This period also saw the first signs of research into the benefits and risks of using analgesic substances, although their use was not yet well understood.^80^ In 1686, the French king Louis XIV underwent surgery on a painful anal fistula performed by the surgeon Charles François Felix de Tassy (1635–1703) using specially designed instruments. Apparently insignificant, this successful intervention allowed medicine to gain the support of the powerful upper class and the further development of new treatments to reduce pain.^41^

Although the secularization of society and the support of rulers were assets for the technological revolution of the time, no marked progress were made until the discovery of the first anesthetics such as nitrous oxide and ether.^16^ The development of analgesic substances proved decisive in reducing pain caused by treatments (including surgeries) and paved the way for centuries of work aimed at developing new pain-relieving drugs.

## 7. Science in the 19th century

Building on the achievements of the Renaissance, the contemporary era continued to make progress in the field of pain. Experimental physiology conducted in France by Claude Bernard (1813–1878) led to significant advances.^6^ *The Lancet*, established in 1823, and *Nature*, founded in 1869, marked the advent of peer-reviewed scientific journals, aiming to expedite the dissemination of science.

### 7.1. The specificity theory

Drawing on Cartesian concepts of pain, the Pain Specificity Theory regained momentum in 1811. Scottish physician Charles Bell (Table 2), whose work focused on the ventral horn of the spinal cord and its motor functions, demonstrated that the ventral and dorsal horns differed in their functions. In 1822, his French contemporary François Magendie (Table 2) described the dorsal horn pathway, stating it essential in the transmission of sensory information.^66,67^ This distinction between motor and sensory pathways is known as the Bell-Magendie law.^40^ In parallel, Johannes Peter Müller (1801–1859) developed the principle of specific energy in nerves (“the law of Specific Sensory Energies”) in 1837. According to this principle, there are as many nerve fibers as there are possible sensory stimuli, and each fiber transmits a single type of message to the brain. Building on these works, Moritz Schiff (1823–1896) developed a new theory of specificity in 1858, stating that pain follows a separate path from other senses. This theory posits that inducing a targeted lesion on the spinal cord yields varying consequences for the sensations touch and pain.^66^ This was supported by the work of Charles-Edouard Brown-Séquard (1817–1894) on animals, and that of William Gowers (1845–1915) in humans, among others. This specificity theory emphasized the involvement of distinct pathways for each somatosensory modality, which would be linked to a specific receptor that responds to a particular stimulus and transmits nerve signals through a distinct pathway.^61,66,67^ The later discoveries of cutaneous receptors, such as the Pacinian corpuscles and Merkel's disks, provided further support for this theory.^61^

### 7.2. The Pattern Theory

In contrast to the specificity theory, the German neurologist Wilhelm H. Erb (1840–1921) proposed in 1874 the notion that any stimulus, when reaching a certain intensity, could ultimately evoke a sensation of pain. This pattern theory spread rapidly. In the late 19th century, Magnus Blix (1849–1904) found that mechanical stimulation on the skin could elicit a painful response; however, when the same stimulation was applied to a nearby area, distinct sensations were experienced.^61,67^ Alfred Goldscheider (Table 2) confirmed these observations in his own work. At the end of the 19th century, Maximilian von Frey (Table 2) conducted a series of experiments related to the work of Blix and Goldscheider. He identified 4 basic somatosensory modalities distributed across the skin: heat, cold, touch, and pain. He also developed the famous von Frey filaments, still used in clinics today, originally made of horsehair. These findings were revolutionary in the field of pain therapeutics. Finally, it is worth mentioning the work of F. W. Sertürner, who developed the pharmacological use of morphine in 1806, after its discovery by the French chemists Seguin and Courtois in 1804. The potency of this molecule led to its easy prescription, and new concerns arose regarding overdose and observed dependence effects.^56^ These concerns are still relevant today, with the opioid crisis affecting several Western countries.^47,69^

### 7.3. The debate continues

David Julius and Ardem Patapoutian, both nominated for the 2021 Nobel Prize in Physiology or Medicine, have revived the debate between specificity and pattern theory. Work by David Julius (University of California, Los Angeles) has highlighted the contribution of highly specific receptors (TRPV1) to warm stimuli in the nociceptive information line.^15^ These data underlined the specificity of nociceptive pathways. Shortly afterwards, Ardem Patapoutian (Scripps Research Institute) discovered channels (Piezo 1 and Piezo 2) sensitive to both painful and nonpainful mechanical deformation,^21^ underlining the nonspecific aspect and patterned activation. Many years after the pioneering work on specificity theory and pattern theory, it was apparent that science, despite major technological advancements, had not come to a definitive resolution. Nevertheless, there was no need to oppose these 2 visions: pain could be a system where both specific and nonspecific processes coexist.

## 8. Modern theories of pain and biopsychosocial approaches

The 20th century marked a turning point in the history of pain, with an acceleration of knowledge. Electrophysiology made key advances, allowing for a better understanding of how sensory information is encoded and transmitted. Charles Sherrington (1857–1952) and Edgar Douglas Adrian (1889–1977) shared the 1932 Nobel Prize in Physiology or Medicine for their respective work on the function of neurons. The Pattern Theory lost influence after Sherrington described specific “receptors” for pain (bare nerve endings), which he called nociceptors, in the early 20th century. Later, Thomas Lewis (1881–1945) at the University of London described specific Aδ and C sensory fibers involved in nociception. William K. Livingston was instrumental in shaping the understanding of pain as we know it today, defining pain as the result of a process involving higher-level cognitive systems. Livingston's work also established a link between the somatic expression of pain and its psychological expression.^65^ In addition to the development of new theories, it is important to highlight the clinical and research contribution of Henry K. Beecher (1904–1976). Beecher was an American anesthesiologist and researcher. His contribution was significant in the development of pain research (clinical trials, placebo effect) and research ethics on humans.^5,60^ He also played an important role in the development of anesthesiology as a medical specialty.^25,60^ Contemporary of H. Beecher, John Joseph Bonica (Table 2) has also left its mark on the history of pain. In 1947, after military service, Bonica became chief of anesthesia at Tacoma General Hospital. There, he initiated the first anesthesia residency in Washington State and developed a major surgical and obstetric anesthesiology service. Bonica published the first textbook of pain management^97^ in 1953 in which he defended the need for multidisciplinary care, a vision universally favored today. He introduced this multidisciplinary vision when he developed the first pain management and treatment clinic, based at the University of Washington Medical Center in Seattle. In 1973, he organized a symposium on pain, laying the foundations for the creation of an interdisciplinary international organization. This organization became in 1974 the International Association for the Study of Pain (IASP), which today has thousands of members worldwide.

The multidimensional (psychological–biological–social) aspect of pain has steadily been developing since the work of Bonica. This biopsychosocial vision had already been applied in the field of mental health.^28^ It is to the anesthesiologist John D. Loeser that we attribute the development of this multidimensional approach to pain,^48,49^ which continues to be used in clinical practice today.

### 8.1. A gate that opens and closes

In 1965, the gate control theory was proposed by Canadian psychologist Ronald Melzack (Table 2) and British physiologist Patrick D. Wall (Table 2). The gate control theory posits that primary afferents transmit sensory information to the superficial layers of the dorsal horn of the spinal cord and that interneurons in these superficial layers act as a gate that modulates nociceptive messages before they are received by supraspinal projection neurons (ie, the spinal cord's output pathway). Large-diameter (Aβ) primary afferent fibers that convey non-nociceptive sensory information tend to close the gate by recruiting spinal inhibitory activity, whereas small-diameter fibers (Aδ and C) that transmit nociceptive information stimulate spinal facilitatory activity.^51,58^ The work by Melzack and Wall underlined for the very first time that pain is not simply the result of nociceptive input and that the nociceptive signal can, on the contrary, be modulated and even inhibited as soon as it enters the spinal cord, at it synapse with the second-order neuron. These observations have had a major impact on pain science, bringing a paradigm shift in our understanding of the underlying mechanisms and our knowledge of treatments. For example, transcutaneous electrical nerve stimulation (TENS) interventions, frequently used in clinical practice, is grounded in gate theory. Later revised in light of findings on spinal cord neuronal networks, the concepts derived from this theory also recognized the influence of supraspinal structures capable of modulating the encoding of nociceptive information.

Subsequent work led by Daniel Le Bars and Tony Dickenson demonstrated that dorsal horn neurons in the spinal cord are inhibited by a nociceptive afferent signal applied to any other part of the body.^44,45^ This modulation, originating from the *raphe magnus,* has been shown to inhibit ascending nociceptive transmission in rats. These studies significantly contributed to the identification of the cerebral structures that exert a descending inhibitory control on nociceptive input. These processes were called diffuse noxious inhibitory controls and were later developed in human models with the popular conditioned pain modulation.

### 8.2. Pain nowadays

Although fundamental research has long focused on the study of peripheral nerves and spinal cord networks, pain is ultimately a consciously processed sensory and emotional experience. Numerous supraspinal structures serve as relays for this elaboration: with advances in brain imaging, these structures have gradually been identified such as sensory (primary and secondary somatosensory cortices), affective (anterior and middle cingulate cortex, insula and prefrontal cortex), cognitive (anterior and middle cingulate cortex, prefrontal cortex, secondary somatosensory cortex), and motor (supplementary motor area, cerebellum) brain regions.^22,34,51^ An activation sequence has also been proposed that allows the extraction of first-, second-, and third-order matrices—originally proposed by Melzack in his Neuromatrix Theory of Pain^57^—associated with the possible encoding of the sensory-discriminative, affective-emotional, and cognitive-behavioral components of pain expression.^30,83^ These findings are consistent with the International Association for the Study of Pain's (IASP) proposal, reinforcing the biopsychosocial vision of pain, as reflected in its most recent definition.^73^ Extracorporeal factors such as past psychological traumas and cultural reality are now considered when assessing the patient.^7^

The World Health Organization now recognizes chronic pain as a disease.^89^ Periodic Global Burden of Disease surveys reveal the worldwide impact of chronic pain on the burden of disease.^35^ The years lived with disability (YLD) indicator expresses the morbidity of a disease in years of life lost because of disability, with chronic low back pain being the leading cause.^76^ In industrialized countries, 1 of every 4 adults experiences chronic pain^10,17,18^; this condition poses a substantial public health challenge and imposes a significant societal burden.

Despite the importance of the problem, pain and its management are still insufficiently addressed. In many countries, only few hours of classes are devoted to pain over 6 years of medical training.^11,46,84,85,94^ Founded in 1974, the International Association for the Study of Pain brings together numerous national chapters and facilitates better coordination to advance the understanding and management of pain. Despite considerable progress in its understanding, many challenges would have to be overcome, including prioritizing the implementation of tools for more accurate pain assessment and enhancing multidisciplinary patient care. In addition, personalized and integrated healthcare, tailored to the individual, need to be coupled with the development of novel therapeutic strategies that merge conventional and integrative medicine, supported by complementary approaches.

## 9. Conclusion

Pain has been ubiquitous in the experience of humankind, shaping learning, culture, beliefs, and understanding of the world of distinct cultures. A crucial insight gleaned from this review is that in the realm of pain, not all advancements revolve solely around 20th-century discoveries. Long before these discoveries, spanning hundreds and thousands of years, a wealth of knowledge existed regarding the comprehension and management of pain. Scientific progress unfolds in a dynamic manner, often exhibiting nonlinear trajectories, with certain areas experiencing exponential growth while others undergo prolonged periods of stagnation. As knowledge is accumulated and disseminated across history, its depth and breadth expand. The proverb “standing on the shoulders of giants” resonates deeply in this context.

Long considered an expression of internal suffering, pain has fascinated humanity throughout history. The way pain has been perceived, understood, and treated has changed greatly over the centuries. Philosophers, priests, physicians, and scientists have tried to understand and make sense of pain. The Greco-Roman era was probably one of the most fruitful periods in philosophical and physiological inquiry, which has since been exported beyond the West and has served as the basis for the development of a new Arabic pain medicine. This new knowledge from the East bridged the gap in scientific progress during the Middle Ages. This historical and scientific review allows us to keep in mind that the vision we have of pain in Western society is predominantly based on the concrete heritage of ancient civilizations, in which Mediterranean cultures have played an important role.

## Disclosures

The authors have no conflict of interest to declare.
